# Oral Health-Related Quality of Life in institutionalized elderly in Barcelona (Spain)

**DOI:** 10.4317/medoral.18280

**Published:** 2013-02-05

**Authors:** Marco Cornejo, Glòria Pérez, Kenio C. de Lima, Elías Casals-Peidro, Carme Borrell

**Affiliations:** 1 DDS, MSc in Public Health. Faculty of Dentistry, University of Chile. - Service of Health Information Systems. Agència de Salut Pública de Barcelona, Spain; 2 MDr, PhD. Agència de Salut Pública de Barcelona - Department of Experimental and Health Sciences. Universitat Pompeu Fabra - CIBER Epidemiología y Salud Pública (CIBERESP), Spain) - Biomedical Research Institute Sant Pau. Barcelona, Spain; 3 DDS, PhD. Federal University of Río Grande do Norte. Brasil; 4 DDS, PhD. Primary Health Care Center Sant Miquel, Barcelona, Spain

## Abstract

Objective: The objective of this study is to describe the oral health status and the factors associated with oral health-related quality of life (OHRQoL) in people aged 65 and older institutionalized in Barcelona in 2009. Study Design: Cross sectional study in 194 elderly. The dependent variable was poor OHRQoL, according to the Geriatric Oral Health Assessment Index (GOHAI). The independent variables were socio-demographic data, last dental visit, subjective and objective oral health status. Robust Poisson regression analysis was used to determine the factors associated with OHRQoL as well as the strengths of association (Prevalence Ratios with respective confidence intervals at 95%). Results: According to GOHAI, 94 women (68.1%) and 36 men (64.3%) had poor OHRQoL. The average DMFT index (number of decayed, missing and filled teeth) was 22.8, with mean 10.2 remaining teeth. According to the Community Periodontal Index only 1.9% were healthy. 33.8% of the sample (35.5% of women and 30.4% of men) presented edentulism, 54.2% needed upper dental prostheses (51.1% of women and 60.7% of men) and 64.7% needed lower ones (61.6% of women and 71.4% of men). Only 7.2% had visited a dentist in the past year (8.8% of women and 3.6% of men). After fitting several multivariate adjusted robust Poisson regression models, poor OHRQoL was found to be associated to self-reporting problems with teeth or gums, self-reporting poor opinion about teeth/gums/denture and also associated to functional edentulism, needing upper denture, but not to socio-demographic factors or time since last dental visit. Conclusions: The study population has poor objective oral health. A high percentage has poor OHRQoL associated to subjective and objective oral health conditions. Dental care is required and these services should be included in the Spanish National Health System.

** Key words:**Oral health, homes for the aged, elderly, self-assessment, quality of life, geriatric oral health assessment index (GOHAI).

## Introduction

The increase in population older than 65 years is a phenomenon with widely discussed social implications in different sectors but particularly in health-related sectors (1). One of the relevant aspects to analyze is oral health in social implications aging population. Both at individual and society levels there is a myth that oral diseases and edentulousness are typical of aging and culturally the image of toothless old people persists. This has been perpetuated because prevention, treatment and recovery of oral health is an issue in which not all communities have defined clear policies (1,2), especially for this group.

On the other hand, aging and oral health should be analyzed taking into account the impact on quality of life. Thus, although historically oral health status was assessed by epidemiological surveys using objective clinical indicators, currently specific measures of oral health-related quality of life (OHRQoL) are also used (3-7). The latter is the subjective interpretation that individuals make of their oral health taking into account previous experiences and the social, economic, cultural and historical context in which they have lived (4), an opinion which is considered important, due to the psychosocial and nutritional implications of oral health problems (8). Gift et al. (9), associate OHRQoL with current issues linked to social determinants of health like external environmental factors such as place of residence (e.g. rural/urban, region or country of origin, etc), access to health care, and individual characteristics such as oral health status, sex, age; and also with self-reported health of teeth, gums and dentures.

Of the different tools for measuring the impact of oral health status on quality of life, the most one commonly used in the elderly, both individually and collectively, has been the Geriatric Oral Health Assessment Index (GOHAI). It has been proved useful as a predictor of the need for an oral examination at individual level (10), since it provides information on symptoms, functional and psycho-social issues in people. Collectively, it can be cost-effective for information on oral health in adult and elderly populations. Purposely, previous studies using GOHAI in Spanish institutionalized elderly populations have reported a high prevalence of poor OHRQoL (5,6).

Spain is a country belonging to European Union where dentistry is mainly private and adult patients must pay the total cost of dental care. One of the benefits offered by public health services is the resolution of dental emergencies, primarily through extrac-tion, but the current portfolio of services excludes recuperative dental care in adults and the elderly (11). However, according to the most current epidemiological data, in the Oral Health Survey - Spain 2010, elderly aged 65-74 show a mean DMFT index 14.7, 16.7% are edentulous, and measured by CPI index 94.2% need periodontal treatment. Additionally, a wide national study which was representative for institutionalized elderly aged 65 and over by 2001, showed a poor oral health status in Spanish elderly (12). On the other hand, in Catalonia (a northeast region of Spain) there are no published studies related to OHRQoL in the elderly nor is there a recent description of oral health status in institutionalized elderly, and the only one published almost two decades ago showed great cumulative damage (Puigdollers A et al. Arch Odontoestom Prev Comunit 1995;11:357-70) alike data from the study in 2001 “Oral health issues of Spanish adults aged 65 and over” (12). Therefore based on adapting the conceptual framework of Gift et al. (9), the aim of this study was to describe the oral health status and the factors associated with OHRQoL in people 65 years and older with a residential profile institutionalized in public social-health centers in the Health Region of Barcelona (Spain) in 2009.

## Material and Methods

-Design and study population: This is a cross-sectional study in people aged 65 or older with a residential profile, institutionalized in public funded and concerted long-stay centers for the elderly in the province of Barcelona (Spain) in 2009.

-Sample and selection of participants: Of the 46 public social-health centers having elderly with residential profiles at the time of the study, 25 of them agreed to participate and allowed us to interview elderly patients at times specifically allocated for data collection. The selection criteria were: aged 65 and older, being institutionalized in a socio-medical center of Barcelona with residential profile, besides the ability to answer the GOHAI participants were required have suitable cognitive conditions according to medical diagnosis.

**Figure 1:**
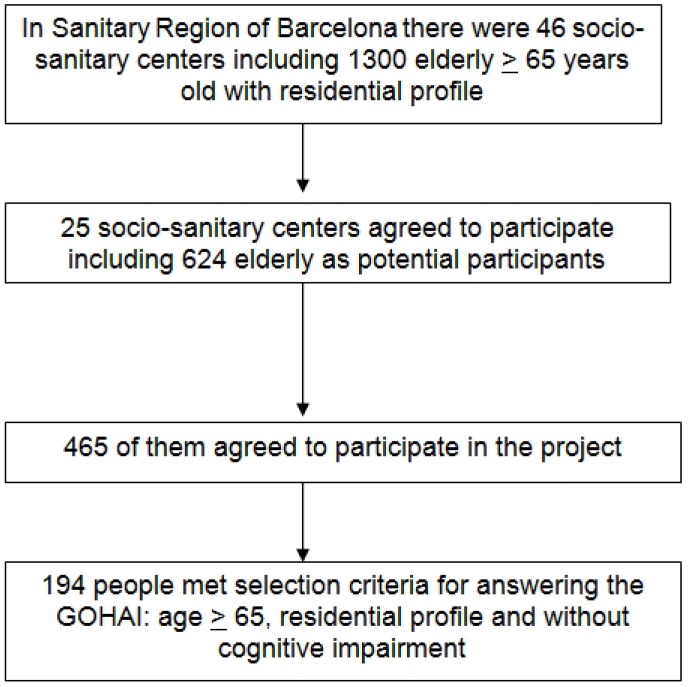
Selection of the sample and the participants.

At the time of fieldwork there were about 1300 people aged 65 and over in the 46 social-health centers. People from those 25 centers that agreed realize the survey on their facilities were 624 elderly as potential participants, that is those that met selection criteria (age and residential profile). 465 elderly agreed to participate in the project to which this study belongs. Of these, only 194 people met the criteria for answering the GOHAI (without cognitive impairment) and constitute the sample used in this study (Fig. 1).

-Fieldwork: data collection was conducted from February to August 2009 by one trained dentist belonging to the research group. During the visit to the centers the dentist performed an oral examination which provided information on clinical oral health and he interviewed the elderly patients in order to obtain data on socio-demographic issues and self-reported oral health, use of dental services and OHRQoL. All participants signed informed consent and this research was approved by the institutional ethical review board of the Hospital del Mar (CEIC-PSMAR).

-Variables and indicators: The dependent variable was OHRQoL categorized according to the score obtained in the GOHAI, using the Spanish version validated for elderly populations, with 12 questions scored from 1 to 5 (total 12 to 60 points) corresponding to worst and best OHRQoL respectively, which we dichotomized into poor (negative) in individuals with a score <57 and good (positive) in those with scores ?57 (cutoff point defined by Pinzón et al. in 1999). The questions cover three dimensions: a) psychosocial (concern about oral health, self-image and limited social contacts due to oral problems), b) physical (eating, speaking and swallowing), c) pain or discomfort associated with oral health.

The independent variables were: a.- Individual characteristics: sex, age grouped in 65 to 74 and ?75 years; educational attainment grouped as “educated” (primary education and over) or “no education” (less than primary education); b.- Oral health behavior: time since last visit to the dentist grouped into 1 year or less, greater than 1 year; c.- External environment: region of origin (Catalonia, another Spanish region, outside Spain); d.- Subjective oral health: subjective oral health conditions (self-reported) such as the existence of any teeth problems (Yes/No), any gum problems (Yes/No) and opinion about their own teeth, gums and/or dentures categorized as Yes (fair, poor or very poor) and No (good or excellent); e.- Objective oral health conditions measured by the dentist: assessed following the criteria of the World Health Organization (WHO) for oral health surveys (13): using upper and lower dentures (Yes/No); needing upper and lower denture as technical and professional criteria (Yes/No), defining prosthetic rehabilitation required if tooth loss affects the aesthetics and/or functionality; needing periodontal treatment according to the Community Periodontal Index (CPI) and sextant of the mouth showing the worst periodontal condition for each individual. CPI index was categorized as needing periodontal treatment: Yes (bleeding, calculus or pockets) / No (healthy people and those with all sextants excluded); Decayed, Missing and Filled teeth (DMFT) index defined as the sum of the number of decayed, missing and filled teeth, and also categorized according to the mean, i.e. ?23 and >23; Edentulism defined as the total absence of natural teeth (Yes/No). Also evaluated “functional edentulism” (the existence of less than 20 teeth).

-Statistical Analysis: descriptive analysis of the variables by sex and bivariate analysis, using the Chi Square test or Fisher’s exact test, were performed to determine the prevalence of poor OHRQoL (GOHAI) by independent variables. Adjusted robust Poisson regression bivariate and multivariate models were fitted to obtain prevalence ratios (PR), and their respective confidence intervals at 95%, to determine factors associated with poor OHRQoL. In the modeling process all variables with p<0.05 for test of association were included, as were certain other variables which were close to statistical significance or had conceptual plausibility. These models where fitted separately for each one of the subjective variables due to the high collinearity between them. Considering the limited sample size, only the univariate analyses were performed stratified by sex. All analyses were conducted using STATA 10.1 for Windows.

## Results

 Table 1 shows the description of the variables by sex and no variable showed statistically significant differences by sex although some differences were observed. Women had a higher prevalence of poor OHRQoL according to GOHAI (68.1%) than men (64.3%). In the sample, more than 74% were older than 75 years. 62.5% of men and 72.5% of women had not completed primary education. Only 3.6% of men and 8.8% of women visited a dentist in the past year and 42% were born in Catalonia. While, related to subjective oral health conditions, 48.1% of men and 43.1% of women self-reported having a poor opinion about their own teeth, gums or dentures. Of those with at least one tooth remaining, 42% self-reported teeth problems. Regarding objective oral health status, wearing dentures was more frequent in women (50.4% upper and 36.8% lower dentures) than men (39.6% and 28.6% respectively), although a greater percentage of men needed them. Over 74% of the sample was “functional edentulous”.

**Table 1:**
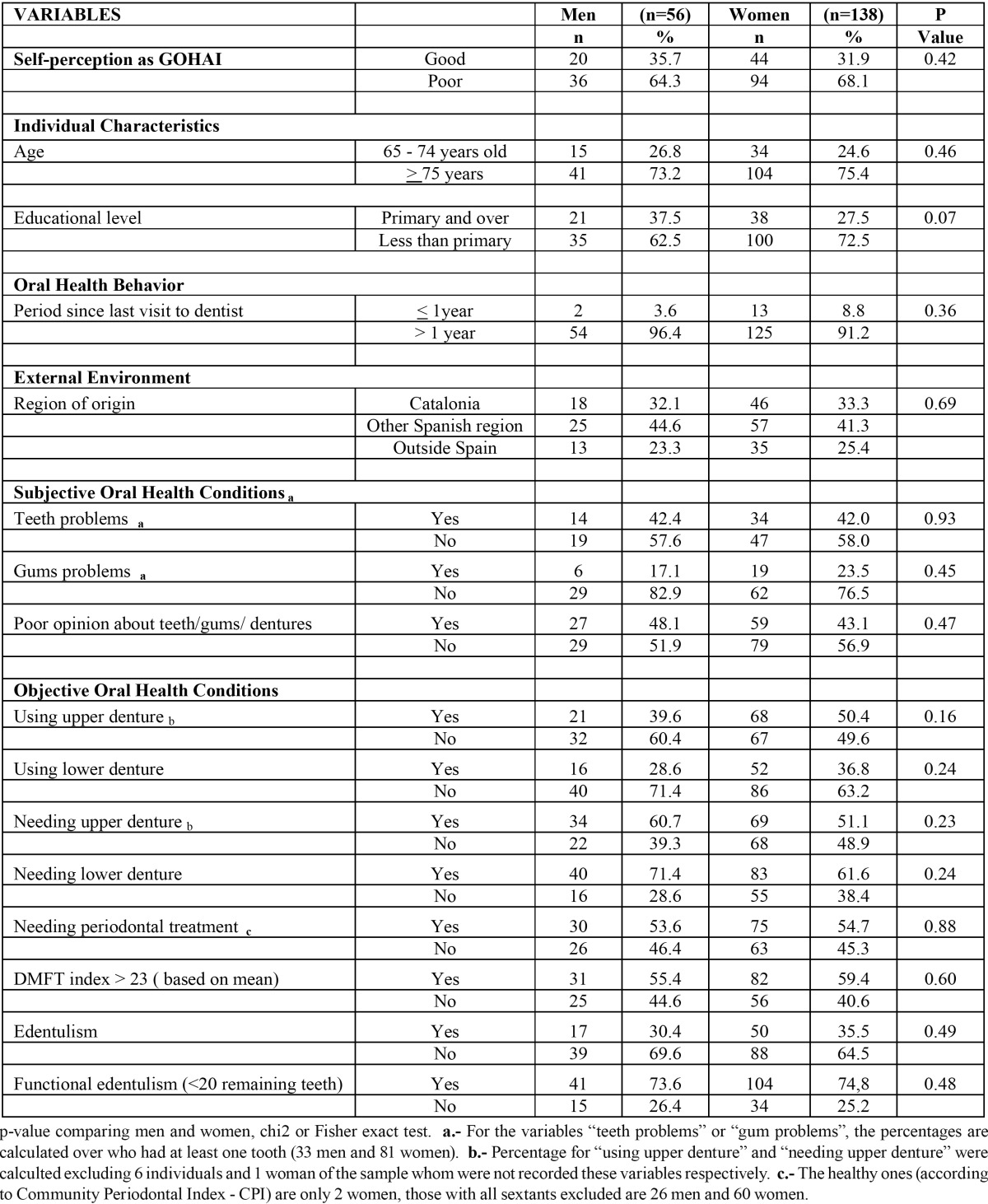
Description of institutionalized men and women with residential profile in geriatric centers in Barcelona (Spain) , 2009.

 Table 2 shows that in terms of periodontal status, assessed using the Community Periodontal Index, 46.4% of men and 43.5% of women had all sextants excluded and of those remaining, 100% of men and 97.4% of women needed periodontal treatment. Regarding DMFT index, the mean was 22.8 in men (95% CI: 19.9 to 25.4) and 23 in women (95% CI: 21.3-24.7). In terms of its components, the average for decayed was 2.2 and 2.0, for missing 20.4 and 20.7; and for filled 0.2 in men and 0.3 in women respectively. The average number of remaining teeth was 10.6 in men and 9.9 in women.

**Table 2:**
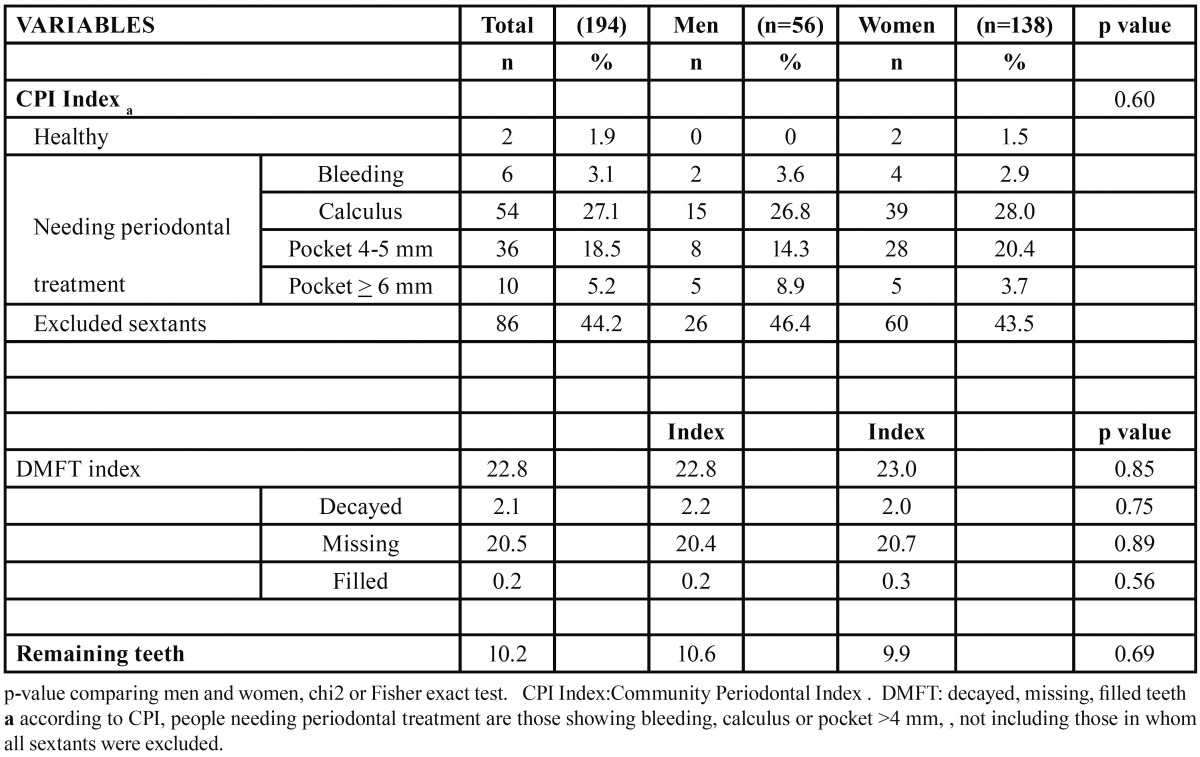
Description of the Community Periodontal Index (CPI) and Decayed, Missing and Filled Teeth Index (DMFT) and number of Remaining teeth of institutionalized elderly with residential in social-health centers in Barcelona, 2009. Total men and women.

Although women, people with higher educational level and those who visited a dentist during the previous year showed a greater prevalence of poor OHRQoL, according to the bivariate analysis no socio-demographic related variables (e.g. sex PR 1.09 95%CI 0.86-1.39), nor last visit to dentist (PR 0.9 95%CI: 0.65-1.24) were statistically significantly associated with poor OHRQoL (GOHAI). All subjective and only some objective oral health conditions (using maxillary denture PR 0.77 95%CI: 0.62-0.96, needing upper prostheses PR 1.44 95%CI:1.15-1.81, and needing lower prostheses PR 1.36 95%CI:1.06-1.75) were found to be associated to poor OHRQoL. Even though “functional edentulism” was not statistically significant in the bivariate analysis (PR 1.26 95%CI:0.96-1.64), it was introduced in multivariate models due to its conceptual plausibility. Variables that explain the poor OHRQoL were “functional edentulism” and needing upper denture, independently when they were adjusted for each one of the subjective variables, except “functional edentulism” which was not statistically significant when adjusted for self-reporting gums problems ( Table 3).

**Table 3:**
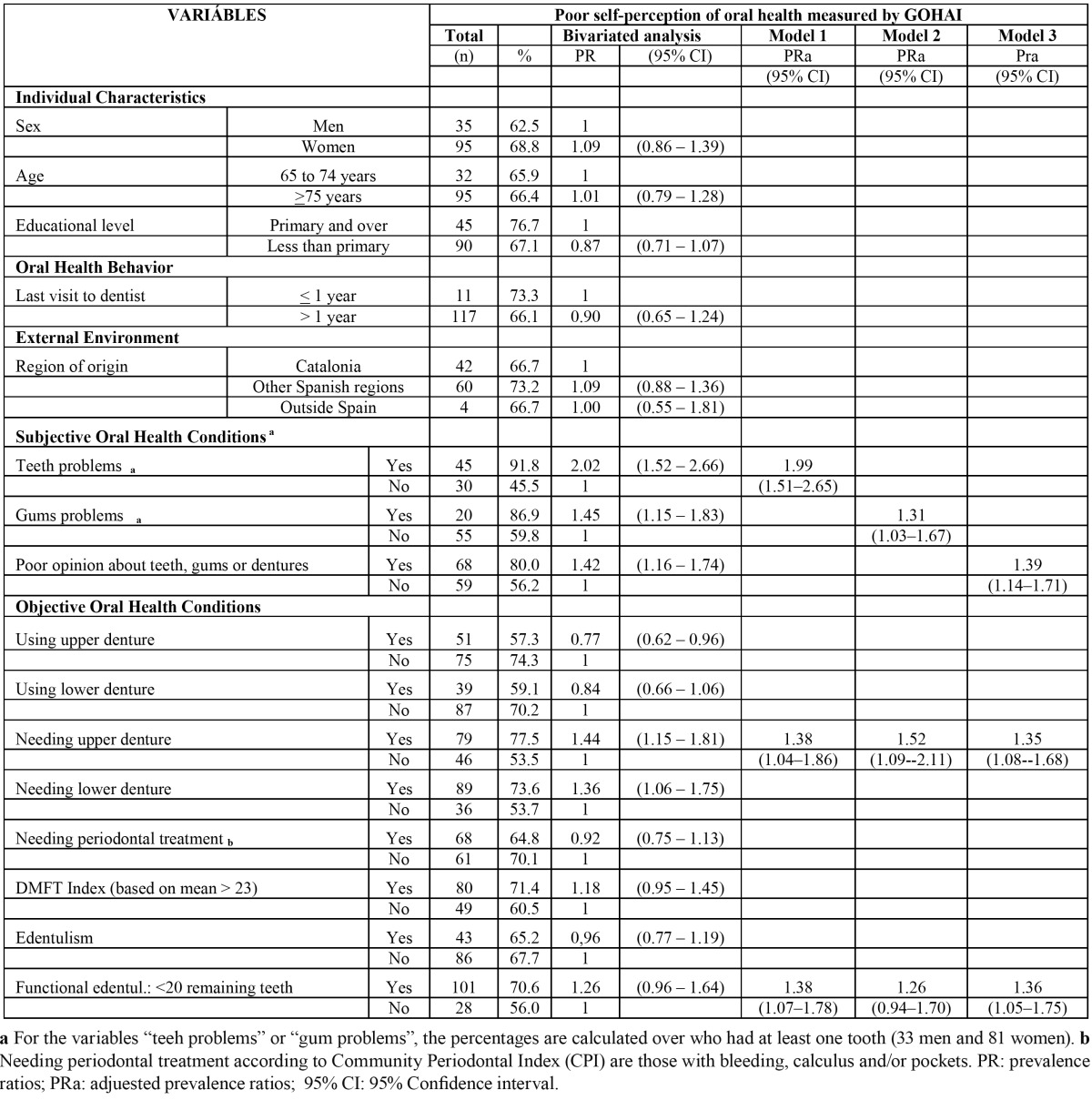
Prevalence (%) of poor oral health self-perception (measured by GOHAI) and factors associated with it. Institutionalized elderly with residential profile in social-health centers in the health region of Barcelona, 2009.

## Discussion

Institutionalized elderly people in Barcelona with a residential profile show high prevalence of poor objective oral health and poor OHRQoL. Subjective oral health conditions, needing upper denture and “functional edentulism” explain the poor OHRQoL. These results are specially interesting considering that almost 75% of the sample is over 74 years old and that Spain, belonging to the European Union, is a country where dental care is not covered by the National Health System.

Prevalence of poor oral health and poor oral health-related quality of life: Our study population shows a high prevalence of poor objective oral health conditions. The prevalence of edentulism (33.8%) was similar to that reported for 2009 by Zuluaga et al. (5), and we found a lower prevalence of edentulism and DMFT index (22.8) in the institutionalized population 65 and older than other studies in Spain (6), and also lower than those reported for catalan institutionalized elderly by 1990 in (Puigdoller A et al. 1995) and for Spanish elderly by 2001 (12). These differences would respond to that those studies have analyzed oral health status for this population during 1990-2001, and their oral health status may have changed in the years which have elapsed since then.

The prevalence of poor OHRQoL is also greater than that described by Zuluaga et al(5) for institutionalized elderly in Granada (which population of study had at least 3 teeth remaining and/or wearing a removable prosthesis), although our results show similar prevalence to that described for this aged population in whole Spain (6). However, several studies using GOHAI show a lower prevalence of poor OHRQoL in institutionalized elderly (4,14-16). The difference between our results and other studies not only correspond to differences in objective oral health conditions but also, as our conceptual model shows (9), to socio-cultural factors, individual history and treatment of oral diseases and access to dental care. Hence many other life events and socially and culturally derived values, also appear to affect an elderly person’s perception of the impact of oral health and oral disease (17). Therefore, to describe the needs in the institutionalized population, in addition to capturing the damage due to caries, periodontal disease, and edentulism, among other oral diseases; it would be necessary to assess their opinion using validated instruments such as GOHAI or OHIP (18), since according to our results only 48.1% of men and 43.1% of women self-reported a poor opinion of teeth, gums or dentures (opinion frequently asked in National Health Surveys in different countries). However, when trying to measure such an opinion using GOHAI, we observe an increase in the prevalence of poor scores and that 80% of elderly self-reporting a poor opinion about their oral health conditions, showed poor OHRQoL (GOHAI).

Otherwise it seems that aesthetic, psychosocial and cultural issues (changing the stereotype of edentulous elderly and giving greater emphasis to a “toothed mouth”), as well as the attention that some administrations and stakeholders would be giving to oral health as part of healthy aging, may have influenced the self-perception of the elderly tested in our study.

Factors related with poor oral health-related quality of life: Regarding the impact of poor OHRQoL, like in other studies it was determined that elderly were needing upper dentures and self-reported poor opinion about their teeth/gums/dentures (4). Also agreeing with Koltermann et al. (19), OHRQoL was associated with “functional edentulism”, as functional-edentulous elders had much worse scores in GOHAI than those with ? 20 remaining teeth. Therefore, our results are consistent with other studies show-ing the importance of clinical oral health status on quality of life for elderly (5,6).

Considering the relation between OHRQoL and oral health status, Matthias et al. proposed to use GOHAI as a predictor of objective oral health conditions in institutionalized elderly (20), with good sensitivity when comparing with other indicators of oral health-related quality of life to identify unmet need for dental treatment (7,10,21). Therefore, using GOHAI to assess the OHRQoL in elderly would contribute to detect and predict the need for dental care, as also to oral health diagnosis and planning of dental services.

Health care utilization, measured as prevalence of elderly who visited a dentist during the previous year, was low compared to that reported for non-institutionalized elderly aged >65 in the Catalan Health Interview Survey 2001-2002 and 2006, and ex-tremely low compared with 38% reported for people >65 in the Oral Health Survey-Spain 2010. But the populations analyzed in the different studies and surveys are not entirely comparable. Although time since last dental visit was not associated to OHRQoL, agreeing with several studies, elderly who visited a dentist the previous year showed a poorer and higher prevalence of poor oral health-related quality of life (4,22). Some authors suggest that the elderly tend to visit a dentist only due to oral problems linked to a painful or uncomfortable experience and also suggest the that decision to visit a dentist would respond to prevailing oral health-related attitudes, dental care phobia linked to bad dental experience, that could influence behaviors like visiting a dentist, leading to a vicious circle that worsens the problems of oral health (4,14,21,23). It is likely that the lack of association reported in our study would also be explained by the very low prevalence of a dental visit during the previous year, maybe also reflecting the lack of access to dental care in Spain (11). Incidentally, the study “Oral Health Survey – Spain 2010” showed that 54% of people aged > 65 think they ought to go to the dentist each year but only 38% actually went.

We must consider that the elderly population has lived in a context in which the loss of teeth and poor oral conditions (reflecting the model of oral health care adopted in many countries, with limited access and uneven, basic and mutilating care), seemed to be considered as normal in aging people. It is likely that lower prevalence of poor OHRQoL reported in other studies, reflects a widespread cultural attitude of resignation (4,9,16), which is less expressed in our study population. That is, there are different perceptions of what is “problematic” according to individual contexts, besides regional and historical tradition, where dental treatment is still poorly accessible, and where it will be more or less likely that a problem was interpreted or perceived as such. We must also consider that clinical indexes tend to measure disease that might be asymptomatic, unknown or not valued by people, while subjective indexes aim to evaluate human and health-related experiences (24). That is, many elderly tend to be considered unhealthy due to acute oral and dental manifestations, but not in cases of chronic irreversible oral processes that lead to tooth loss and even edentulism, a situation in which people tend to believe that their mouths do not need oral health care (4). Even in countries with specific programs for this group, the main reason for not seeking dental treatment is the failure to perceive the need for dental care (23).

Strengths and limitations: A limitation of this study is the small number of people who responded to the GOHAI, which did not allow sex-stratified analyzes and means the statistical power to find gender differences is low. However, the sample size is still larger than the average in most studies using GOHAI in institutionalized elderly. Also, the selection criteria for long-stay residences must be considered when generalizing the results, even though this study is particularly interesting considering that it is the first in Catalonia-Spain using GOHAI in a diverse sample of long-stay residences for institutionalized elderly. Also, more than 74% of the sample were aged over 74 years, an age-group that is traditionally not included in epidemiological studies proposed by the WHO and about whom there is only limited information.

Conclusion and recommendations: People 65 years and older institutionalized with residential profile in Barcelona, show high prevalence of poor objective oral health status (CPI and DMFT indexes, “functional edentulism”, etc) and unmet needs for dental care that were associated with poor OHRQoL. The oral health status of this population and the accumulation of oral health damage requires that dental care coverage by the National Health System be increased, to tackle the impact of oral heatlh and oral health-related quality of life. Therefore, it is necessary to improve health and social importance of oral health and oral health care for elderly, aspects that become relevant in relation to healthy aging and human rights issues in the discussion about economic efficiency and healthcare spending (25). Moreover, in recent years in most of the developed countries a very important trend to keep teeth has appeared, so the impact of diseases such as caries (restorative dentistry) or gum disease (periodontal care) is decisive when making assessments of the preventive and curative care needs of this population. Thus it is necessary to include oral health care in the portfolio of services in the National Health System, specially to contribute to the oral health goal for Spain-2020 and to achieve 15% or less of edentulism in people 65 to 74 years old.

## References

[1] Listl S (2011). Income-related inequalities in dental service utilization by Europeans Aged 50+. J Dent Res.

[2] Barriuso Lapresa L, Sanz Barbero B (2011). Multilevel analysis of the use of oral health services by the pediatric population. Gac Sanit.

[3] Llewellyn CD, Warnakulasuriya S (2003). The impact of stomatological disease on oral health-related quality of life. Eur J Oral Sci.

[4] Martins AMEB, Barreto SM, Pordeus IA (2009). Objective and subjective factors related to self-rated oral health among the elderly. Cad Saude Publica.

[5] Zuluaga DJM, Montoya JAG, Contreras CI, Herrera RR (2012). Association between oral health, cognitive impairment and oral health-related quality of life. Gerodontology.

[6] Gil-Montoya JA, SubirÃ C, RamÃn JM, GonzÃlez-Moles MA (2008). Oral health-related quality of life and nutritional status. J Public Health Dent.

[7] Hassel AJ, Rolko C, Koke U, Leisen J, Rammelsberg P (2008). A German version of the GOHAI. Community Dent Oral Epidemiol.

[8] Polzer I, Schimmel M, MÃller F, Biffar R (2010). Edentulism as part of the general health problems of elderly adults. Int Dent J.

[9] Gift HC, Atchison KA, Drury TF (1998). Perceptions of the natural dentition in the context of multiple variables. J Dent Res.

[10] SÃnchez-GarcÃa S, Heredia-Ponce E, JuÃrez-Cedillo T, Gallegos-Carrillo K, Espinel-BermÃdez C, De La Fuente-HernÃndez J (2010). Psychometric properties of the General Oral Health Assessment Index (GOHAI) and dental status of an elderly Mexican population. J Public Health Dent.

[11] Bravo M, CortÃs J, Casals E, Llena C, Almerich-Silla JM, Cuenca E (2009). Basic oral health goals for Spain 2015/2020. Int Dent J.

[12] Spanish Geriatric Oral Health Research Group (2001). Oral health issues of Spanish adults aged 65 and over. The Spanish Geriatric Oral Health Research Group. Int Dent J.

[13] (1997). Oral Health Surveys: Basic Methods).

[14] Benyamini Y, Leventhal H, Leventhal EA (2004 9). Self-rated oral health as an independent predictor of self-rated general health, self-esteem and life satisfaction. Soc Sci Med.

[15] Locker D, Clarke M, Payne B (2000). Self-perceived oral health status, psychological well-being, and life satisfaction in an older adult population. J Dent Res.

[16] Kressin NR, Atchison KA, Miller DR (1997). Comparing the impact of oral disease in two populations of older adults: Application of the geriatric oral health assessment index. J Public Health Dent.

[17] McMillan AS, Wong MC, Lo EC, Allen PF (2003). The impact of oral disease among the institutionalized and non-institutionalized elderly in Hong Kong. J Oral Rehabil.

[18] Montero-MartÃn J, Bravo-PÃrez M, Albaladejo-MartÃnez A, HernÃndez-MartÃn L, Rosel-Gallardo E (2009). Validation the Oral Health Impact Profile (OHIP-14sp) for adults in Spain. Med Oral Patol Oral Cir Bucal.

[19] Koltermann AP, Giordani JM, Pattussi MP (2011). The association between individual and contextual factors and functional dentition status among adults in Rio Grande do Sul State, Brazil: a multilevel study. Cad Saude Publica.

[20] Matthias RE, Atchison KA, Schweitzer SO, Lubben JE, Mayer-Oakes A, Jong FD (1993). Comparisons between dentist ratings and self-ratings of dental appearance in an elderly population. Spec Care Dentist.

[21] Dolan TA, Atchison K, Huynh TN (2005). Access to dental care among older adults in the United States. J Dent Educ.

[22] Mesas AE, De Andrade SM, Cabrera MA (2008). Factors associated with negative self-perception of oral health among elderly people in a Brazilian community. Gerodontology.

[23] Kiyak HA, Reichmuth M (2005). Barriers to and enablers of older adults' use of dental services. J Dent Educ.

[24] Locker D, Slade G (1994). Association between clinical and subjective indicators of oral health status in an older adult population. Gerodontology.

[25] MacEntee M (2006). Missing links in oral health care for frail elderly people. J Can Dent Assoc.

